# Study on the mechanical and aging properties of an antibacterial composite resin loaded with fluoride-doped nano-zirconia fillers

**DOI:** 10.3389/fbioe.2024.1397459

**Published:** 2024-05-23

**Authors:** Liyuan Zheng, Yi Zhang, Yuming Bai, Zhisheng Zhang, Qianju Wu

**Affiliations:** ^1^ Department of Prosthodontics, Stomatological Hospital of Xiamen Medical College, Xiamen Key Laboratory of Stomatological Disease Diagnosis and Treatment, Xiamen, China; ^2^ Department of Orthodontics, Stomatological Hospital of Xiamen Medical College, Xiamen Key Laboratory of Stomatological Disease Diagnosis and Treatment, Xiamen, China

**Keywords:** composite resin, nano-zirconia fillers, fluoride-releasing, antibacterial property, aging test, mechanical performance

## Abstract

Preventing the occurrence of secondary caries serves as one of the significant issues in dental clinic, thus make it indispensable to improving the properties of conventional composite resin (CR) by developing a novel CR. In present study, two groups of experimental CRs loaded with different contents of fluoride-doped nano-zirconia fillers (25 wt% and 50 wt%) were fabricated. The surface topography, mechanical performance, fluoride release, antibacterial effect, aging property and cytotoxicity of the experimental CRs were evaluated subsequently. A uniform distribution of the F-zirconia fillers over the whole surface of resin matrix could be observed. The experimental CRs showed continuous fluoride release within 28 days, which was positively correlated with the content of F-zirconia fillers. Moreover, the amount of fluoride release increased in the acidic buffer. Addition of F-zirconia fillers could improve the color stability, wear resistance and microhardness of the experimental CRs, without reducing the flexure strength. Furtherly, the fluoride ions released continuously from the experimental CRs resulted in effective contact and antibacterial properties, while they showed no cytotoxicity. As a consequence, considerations can be made to employ this new kind of composite resin loaded with fluoride-doped nano-zirconia fillers to meet clinical requirements when the antimicrobial benefits are desired.

## 1 Introduction

With the enhancement of aesthetic requirements and the development of novel dental materials, tooth-colored materials like composite resins (CRs) have been widely used in dentistry. Due to their strong mechanical properties and good aesthetic characteristics, CRs have gradually become the most commonly used filling and adhesive materials in clinic, surpassing the use of traditional silver amalgam and the glass ionomer cement (GIC) (Pizzolotto and Moraes, 2022; Aldowsari et al., 2023; Maletin et al., 2023). However, the occurrence of secondary caries at the edge of the CR restorations has become the main cause of secondary filling treatments (Chladek et al., 2023). Furthermore, the secondary filling operations often produce more damage to the hard tissues of teeth, as well as more thermal or chemical stimulus to the dental pulps, which may also lead to the filling failures (Bakhsh et al., 2023). It was reported that the possibility of secondary caries occurring around the CRs was even greater than that using other filling materials such as the GIC or the amalgam (Opdam et al., 2010; Eltahlah et al., 2018). Thus, secondary caries has become an urgent problem in dental clinic. The replacement of CRs has undoubtedly become a heavy burden on the expenditure on health.

Moreover, the polymerization shrinkage occurring in the curing procedure also makes the application of CRs in posterior teeth more stressful, especially in repairing the extensive dental defects (Loguercio et al., 2023). In order to improve the defects of CRs, studies have been conducted to reduce the polymerization shrinkage by improving the filling technique, using the bonding agents in combination (Kaur et al., 2015; Kim et al., 2022; Hosaka et al., 2023). However, the complexity of filling procedure may not only increase the operating time but also improve the technical sensitivity (Shibasaki et al., 2017; Van Ende et al., 2017). Hitherto, there are still no new materials or new technologies that can avoid the microleakage occurring at the edge of the CRs completely. On the other hand, as far as we know that bacterial infection is considered the main cause of secondary caries after restoration, yet the specific cariogenic bacteria and the pathological process are not clear. Thus, adjusting the compositions to develop novel CRs with antibacterial properties has also become one of the research hotspots currently (Stewart and Biostable, 2019).

Back to the early 20th century, the application of fluoride agents had become an important way to maintain oral health (Pollick, 2018). The anti-caries property of fluoride agents is mainly achieved by maintaining a certain concentration of fluoride ions in saliva locally, which forms a mineralization system with calcium ions and phosphate ions together. Calcium fluoride and fluorapatite generated can inhibit the demineralization and promote remineralization process. Furthermore, fluoride can also act on the acid-producing bacteria such as mutans streptococci (S. mutans) directly. While blocking the functions of enzymes related to glycolysis and cellular oxidation, fluoride also inhibits the intake of glucose by the bacteria. Fluoride has been proved to play an excellent anti-caries property in the traditional GIC, and has also been tried to be used in the development of novel antibacterial CRs nowadays (Francois et al., 2020). Since the 1980s, fluoride-containing CRs have been used in the orthodontic adhesives and the fissure sealants, while there were few studies on them as direct filling materials (Ei et al., 2018). But it is worth noting that the release of fluoride ions may result in the porous structure of the material, which will weaken the mechanical properties and wear resistance (Van der Laan et al., 2019).

In recent years, nano-zirconia powders have been applied as inorganic fillers to enhance the mechanical properties of CRs. On the one hand, as one of the commonly used reinforcing materials in biomedicine, zirconia presents excellent strength and biocompatibility. On the other hand, addition of nanoparticles is conducive to increase the aesthetic performance and wear resistance of materials. Furthermore, it was suggested that nanoparticles possess stronger prevention in bacterial adhesion and biofilm formation due to their large surface-volume ratio (Melo et al., 2013). Some commercial CRs products contained nano-zirconia fillers have been used in clinic so far, yet few studies focused on their antibacterial property. Thus, we proposed a hypothesis that combination fluoride with nano-zirconia through a certain way in the fillers of a novel CR would do help to maintain the clinical performance while exerting good fluoride-releasing property. In the early stage of our research, ammonium zirconium hexafluoride was added into zirconium salt as the source of fluoride ions. A kind of high-purity fluoride-doped nano-zirconia particles were synthesized through chemical precipitation and the calcination procedure. A series of experiments were carried out to prove that both the nanoparticles and the novel CR loaded with fluoride-doped nano-zirconia fillers possessed definite release of fluoride ions, and the released ions could inhibit the growth of S. mutans effectively (Zheng et al., 2021).

In the present work, the composition of fillers was optimized furtherly according to the preliminary results. Fluoride release from the experimental CR into media with different pH values were measured to simulate the caries processes. The action mode of antibacterial effect was explored here. Furthermore, thermocycling test were used to simulate the intraoral aging process of the experimental CR. The mechanical performance and cytotoxicity were also evaluated to verify its clinical application prospects.

## 2 Materials and methods

### 2.1 Synthesis of the CRs loaded fluoride-doped nano-zirconia fillers

Fluoride-doped nano-zirconia particles were coated with the silane coupling agent KH-570 to improve the interfacial combination between the fillers and resin matrix. The resin matrix consisted of a 70:30 (w/w) bisphenol-A glycidyl dimethacrylate (Bis-GMA) and tri-ethylene glycol dimethacrylate (TEGDMA). The initiator system consisted of 0.5 wt% camphorquinone (CQ) and 1 wt% ethoxylated bisphenol A dimethacrylate (DMAEMA). The experimental CRs were formulated with the resin matrix and varying concentrations of the silaned fillers (25, 50 wt%) through *in situ* dispersion method. The mixtures were filled to Teflon molds and light cured for 40 s on both sides (1200mW/cm2, Elipar S10, 3M Espe, Seefeld, Germany). For comparison, specimens of the pure resin matrix without fillers were also prepared in the same way.

### 2.2 Field emission scanning electron microscopy

One specimen of each group of the experimental CRs selected and coated with electrically-conductive material. The surface morphology and the dispersion of nanoparticles in CRs were investigated by Field emission scanning electron microscopy (FESEM, MERLIN, ZEISS, Germany).

### 2.3 Mechanical properties

#### 2.3.1 Flexure strength

Specimens of size 25 mm × 2 mm × 2 mm were prepared in each group (N = 5). Before the test, every specimen was polished sequentially using #800, #1000, #2000, and #3000 abrasive papers and then stored in the saline solution at 37 °C for 24 h. After drying thoroughly, the specimens were placed on the universal mechanical testing machine (Instron 5566, Instron, United Kingdom) for flexure strength test at a span length of 20 mm and a speed of 0.5 mm/min. At the same time, the fragmentation load was recorded and the flexure strength was calculated according to the formula: FS = 3Fl/2bh^2^ [Where FS means the flexure strength (unit: MPa), F means the fragmentation load (unit: N), l means the span length, b and h mean the width and height of specimen (unit: mm)].

#### 2.3.2 Wear resistance

Cylindrical specimens with a diameter of 10 mm and a height of 5 mm were prepared (N = 3) and polished sequentially. After stored in the saline solution at 37 °C for 24 h and dried, each specimen was fixed on the mechanical reciprocating friction and wear device (MWF-02, sdbaohang machinery manufacturing co., ltd., China). A Co-Cr stainless steel ball with a diameter of 5 mm was used as the grinding part, and the abradant was prepared by mixing 4:1 (w/w) fluorite powder and water. A 30 N loading force was applied to the specimens during 6000 friction cycles, and the speed of the reciprocating friction movement was 300 rpm/min. The digital micrometer was used to measure the height of specimens before and after the test. Moreover, one specimen was selected randomly to observe the microscopic morphology of the wear surface under SEM.

### 2.4 Fluoride release

Fluoride release of the experimental CRs into various storage media with different pH values during 28 days was determined using a pH meter (a-AB41PH ZH, OHAUS, China) with fluoride ion selective electrode (F, STISE22, OHAUS, China). Three specimens of each group were eluted in either 2 mL distilled water, 2 mL acidic buffer (20 mM KCl, 154 mM NaCl, 3.6 mM NaH_2_PO_4_·H_2_O, pH 4.2) or 2 mL neutral buffer (1.9 mM CaCl_2_, 30mM KCl, HEPES, pH 7.0). All samples were stored in a shaker at 37 °C. The extraction media were collected in the 1st, 3rd, 7th, 14th and 28th days and exchanged by fresh storage media at the time of each measurement. The fluoride ions concentration in the extraction media was measured. Before determination of the samples, the selective electrode was fully activated and calibrated with a series of fluoride standards ranging from 0.001 ppm to 100 ppm.

### 2.5 Antibacterial property

*Streptococcus* mutans (S.mutans UA159, Guangdong Microbial Culture Collection Center, China), which was considered as the primary cariogenic bacteria, was used as the experimental strain. The frozen strain was revived and incubated in sterile Brain Heart Infusion broth-agar (BHI-agar, BD, United States) media under anaerobic condition at 37 °C. A single bacteria colony was selected and transferred into fresh BHI media before the experiment. The microplate reader (Elx800, BioTek, United States) was used to regulate and control the absorbance of S. mutans strain at the wavelength of 600 nm.

#### 2.5.1 Colony-forming units (CFUs) counting

Specimens with 8 mm in diameter and 2 mm thick of each group were prepared (N = 3) and sterilized by ultraviolet light for 2 h before the antibacterial test. The specimens of Resin without the experimental fillers were prepared and taken as control group. All specimens were placed in a sterile 48-well plate with 500 uL fresh BHI media and 50 uL of bacterial suspension (1 × 10^6^ CFU/mL). After incubated for 24 h at 37 °C, the bacteria grow and form biofilms on the surface of specimens. Then, the specimens were transferred to another 48-well plate and washed gently with PBS to get rid of the planktonic bacteria. The biofilm formed on the surface of each specimen was collected by shaking and washing with 500 uL fresh BHI media. And the planktonic bacteria in the original 48-well plate were also collected and mixed well. All experimental bacteria suspensions were diluted to 10-5 times and inoculated on BHI-agar plates to count the bacteria colonies. The antibacterial rate (AR) was determined via the following formula: AR (%) = (C0-C)/C0 × 100% (Where C0 means the number of colonies in average of the control group, C means the number of colonies in average of the experimental group.)

#### 2.5.2 Metabolic activity test

The metabolic activities of both planktonic bacteria and biofilm were estimated by the Cell Counting Kit-8 assay (CCK-8, Dojindo, Japan). After co-culturing with the experimental CRs for 24 h, the planktonic bacteria and biofilm were collected and transferred to a new 48-well plate by the method as above-mentioned in 2.5.1. 50 uL of CCK-8 liquid was added into each well and incubated for another 2 h in the incubator at 37 °C. Finally, the absorbance at 450 nm was determined by the microplate reader (Elx800, BioTek, United States).

### 2.6 Aging test

#### 2.6.1 Thermal aging procedure

Specimens with 8 mm in diameter and 2 mm thick of each group were prepared (N = 3) for thermal aging test. No thermal aging status was denoted as time T0. Then the specimens were subjected to 10,000 cycles of thermocycling between 5 °C and 55 °C with a transfer time of 30 s, which was applied to simulate the thermal aging in 1 year. The time at which the 10,000 cycles ended was denoted as T1.

#### 2.6.2 Color change

Color values of the specimens were measured at T0 and T1, respectively, using a tooth color comparator (VITA Easyshade V, VITA Zahnfabrik H. Rauter GmbH and Co. KG, Germany) in 3-point measurement mode. The color comparator was recalibrated before each measurement. After all measurements, average of the CIELab values (L*a*b*) was calculated for each specimen. And the ΔE00 value was calculated with the online color calculator (CIEDE2000 color system, www.colormine.org) to show the color change from before to after the thermo aging procedure.

#### 2.6.3 Microhardness test

The microhardness values of all specimens were measured using a microhardness (Vickers) testing device (Micro Hardness Tester HMV-G-FA, SHIMADZU, Japan) at T0 and T1. A load of HV0.1 (980.7 mN) was applied to the surface of each specimen in 3 points selected randomly for 10 s. And averages of the microhardness values before and after thermal aging procedure were calculated.

### 2.7 Cytotoxicity

Specimens with 8 mm in diameter and 2 mm thick of each group were prepared (N = 5) and sterilized with 75% ethanol for three times. Each specimen was immersed into Dulbecco’s modified Eagle’s medium (DMEM) high glucose medium (HyClone, United States), which contained 10% fetal bovine serum (Gibco, United States) and 1% pecicillin-streptomycin (EveryGreen, China). Then, the eluates were collected after the incubation at 37 °C for 3 days. A total of human dental pulp cells (HDPs) was seeded per well in a 96-well plate and cultured with DMEM media. After incubated at 37 °C for 24 h, the media was replaced with the eluates of each group. Cells incubated in the pure media without eluates were used as the negative control. The alamar blue kit (Invitrogen, United States) was used for cytotoxicity test according to the manufacturer’s instruction. Fluorescence intensities of HDPs at 1, 3, 5 and 7 days in response to the eluates of experimental CRs were measured using the microplate reader.

### 2.8 Statistical analysis

The data obtained were analyzed with SPSS Statistical software (version 25.0, IBM, Armonk, United States). One-way analysis of variance (ANOVA) and Post hoc analysis for multiple comparisons were performed. *p* < 0.05 was considered as statistically significant.

## 3 Results

### 3.1 Field emission scanning electron microscopy

**FIGURE 1:**
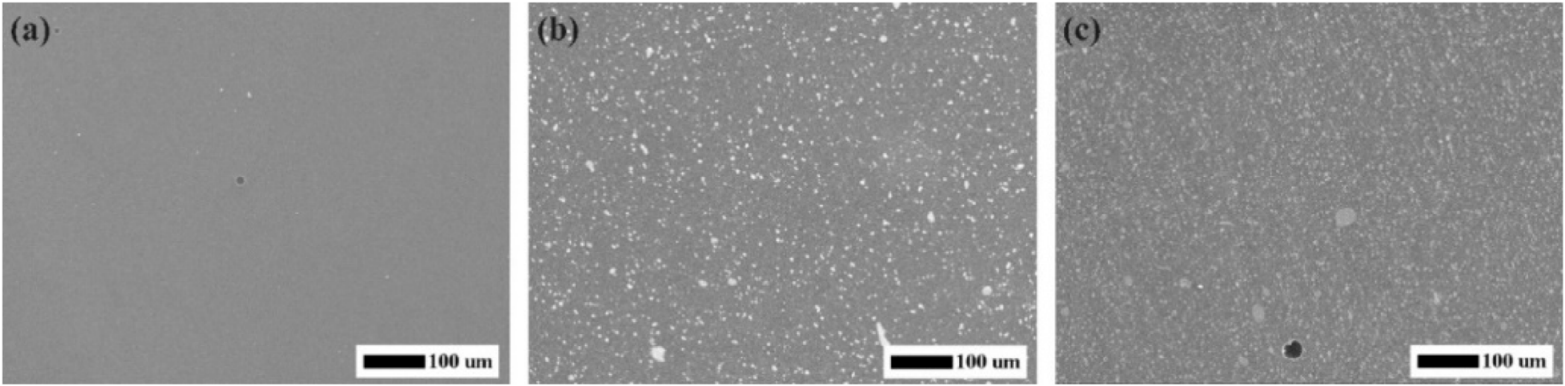
SEM micrographs of the experimental composite resins with fluoride-doped nano-zirconia fillers. **(A)** Control group; **(B)** 25 wt% F-zirconia fillers; **(C)** 50 wt% F-zirconia fillers.

Figure 1 showed the SEM micrographs of the experimental CRs with fluoride-doped nano-zirconia fillers. The micrograph of control group (pure resin matrix) was uniformly dark (Figure 1A), while inorganic fillers presented as gray or white particles distributed uniformly in the resin matrix in the micrographs of experimental CRs (Figures 1B,C). Generally, all the experimental fillers were evenly dispersed in the monomer matrixes and there was only a small minority of agglomeration of fillers.

### 3.2 Mechanical properties

The result of flexure strength (FS) test was shown in Table 1. It could be seen that the FS of control group was the highest, reaching 88 MPa in average. With the addition of inorganic fillers, the FS of specimens showed no significant decline. Statistical analysis of the result showed that there was no significant difference among the three groups (*p* ≥ 0.05). In addition, height loss of the experimental composite resins with fluoride-doped nano-zirconia fillers after the wear resistance test were also presented in Table 1. After the wear resistance test, specimens of the control groups showed a significant height loss of 161 um in average. The height loss decreased to 115 um in average with the addition of 25 wt% F-zirconia fillers, and it furtherly decreased to 60 um in average with the addition of 50 wt% F-zirconia fillers. Statistical results showed that the difference between the group of 50 wt% F-zirconia fillers and the control group was statistically significance (*p* < 0.05).

**TABLE 1:** Flexure strength and height loss after wear resistance test of the experimental composite resin with fluoride-doped nano-zirconia fillers.

Group	Flexure strength (mean ± SD, MPa)	Height loss after wear resistance test (mean ± SD, um)
Control group	88 ± 5.21	161 ± 38.84
25 wt% F-zirconia fillers	82 ± 5.75	115 ± 24.25
50 wt% F-zirconia fillers	85 ± 4.84	60 ± 26.23*

Note: The superscript symbol * means statistical difference compared to the control group (*p* < 0.05).

**FIGURE 2:**
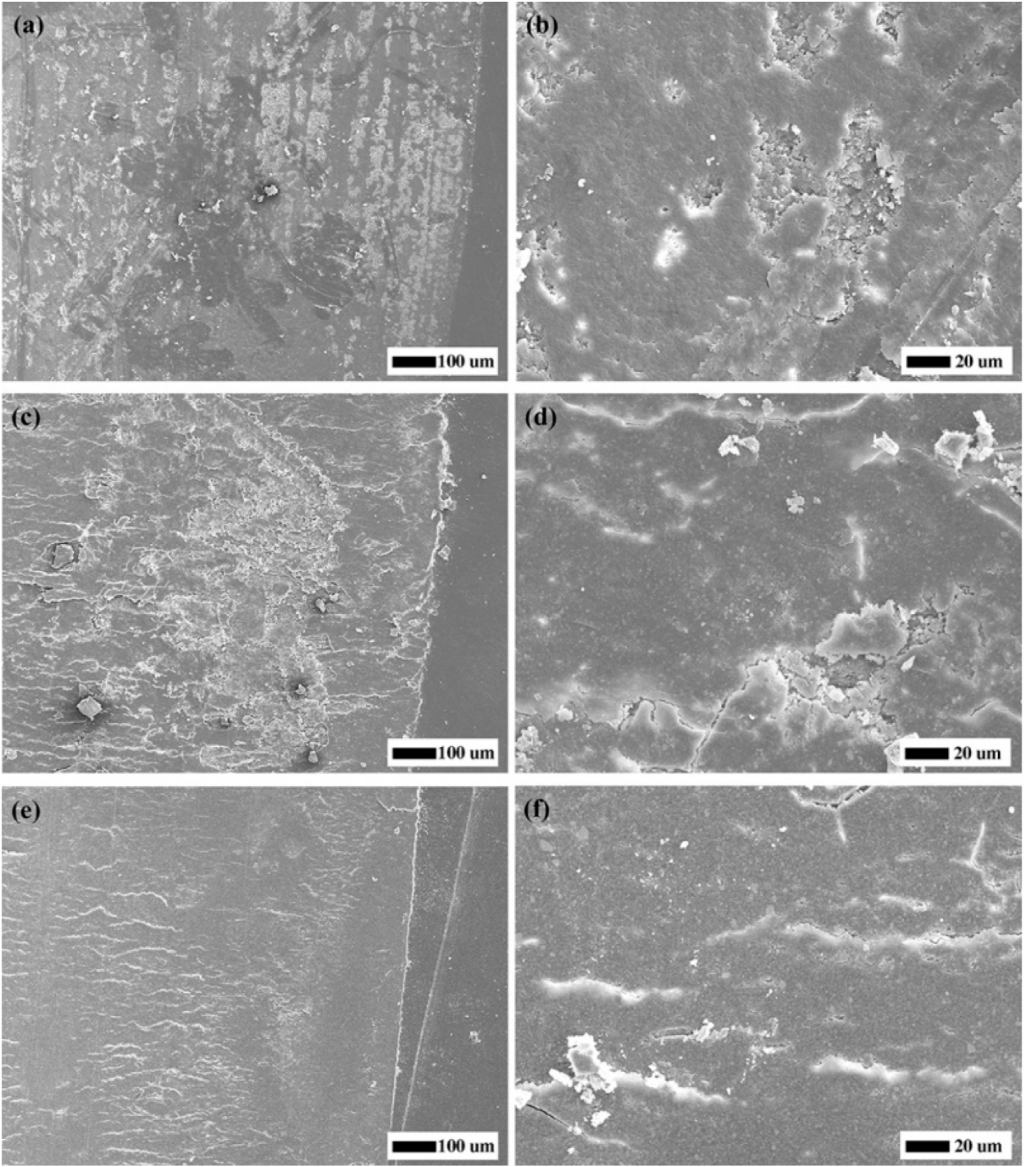
SEM micrographs of worn surfaces of the specimens. **(A,B)** Control group; **(C,D)** 25 wt% F-zirconia fillers; **(E,F)** 50 wt% F-zirconia fillers.

The morphologies of worn surfaces after the wear resistance test were analyzed via SEM and presented in Figure 2. It could be seen that there were obvious longitudinal wear traces and exfoliative pits on the wear surface of the control group at low magnification (Figure 2A). At high magnification, irregular pits could be found after the outermost layers of resin falling off (Figure 2B). For the group of 25 wt% F-zirconia fillers, horizontal wear traces were observed on the surface, and scattered agglomerated particles were exposed at low magnification (Figure 2C). Further, cracks and small pits appeared after the fillers falling off at high magnification (Figure 2D). Similarly, the wear surface of 50 wt% F-zirconia fillers after wear resistance test was relatively flat and the wear traces were horizontal under low magnification (Figure 2E). Friction striations could be seen at high magnification, a small number of fillers were exposed and scattered, while there were no obvious stripping pits (Figure 2F).

### 3.3 Fluoride release

**FIGURE 3:**
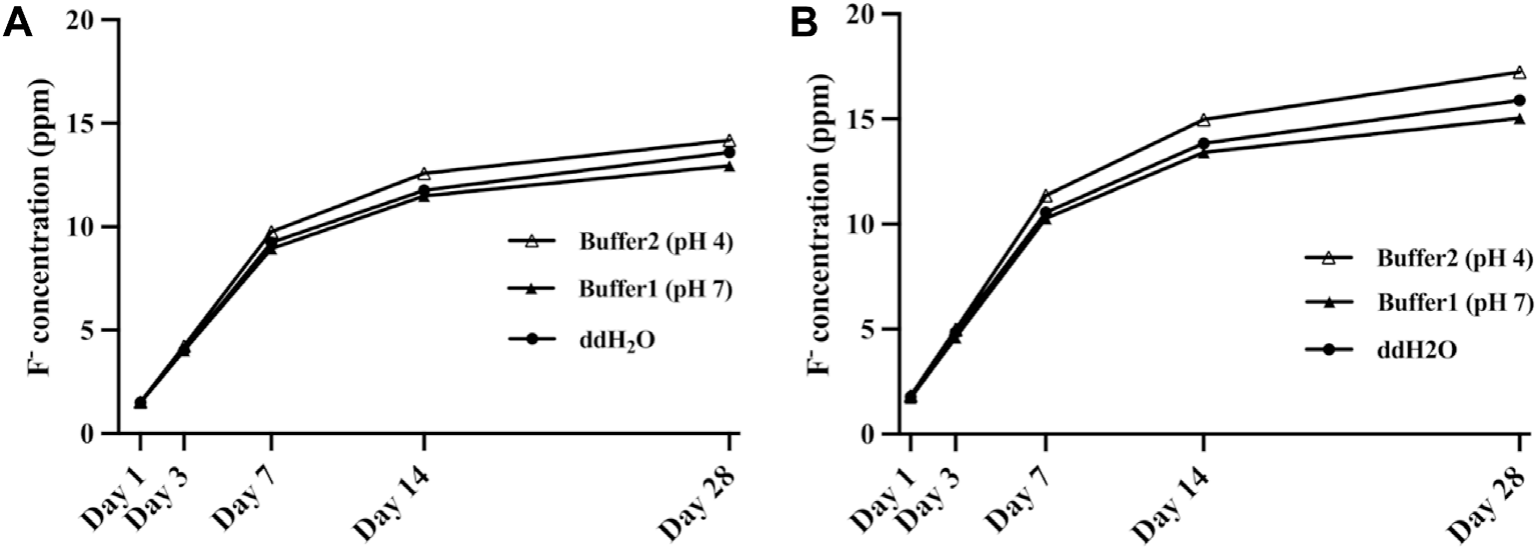
Cumulative fluoride release of the experimental composite resin with fluoride-doped nano-zirconia fillers in different buffers within 28 days [**(A)** 25 wt% F-zirconia fillers; **(B)** 50 wt% F-zirconia fillers].

Table 2 and Table 3 showed the daily and cumulative amount of fluoride release from the experimental CRs within 28 days, respectively One-way ANOVA test revealed that both the quantity of fillers and time had a statistically significant effect on the amount of fluoride release. In general, the cumulative amount of fluoride released from 50 wt% F-zirconia fillers was significantly higher than that from the group of 25 wt% F-zirconia fillers (*p* < 0.05). Regarding detection of the daily amount, the fluoride release decreased obviously with time. The difference was statistically significant in neutral buffer in every detection point (*p* < 0.05). And the difference between the two groups became more obvious in day 14 and day 28 (*p* < 0.05). However, the pH of media had a little effect on fluoride release. The amount of fluoride release increased in the acidic buffer than that in another two media, but the difference was not statistically different (*p* ≥ 0.05). The change trends of cumulative fluoride release could be observed in Figure 3. For both two groups of the experimental CRs, a significant upward trend could be seen in the beginning 7 days, while the upward trend had slowed over the remaining observation period.

**TABLE 2:** Daily fluoride release (ppm) from the experimental composite resin with fluoride-doped nano-zirconia fillers in different buffers during 28 days.

Time	Groups	Distilled water mean (SD)	Neutral buffer mean (SD)	Acidic buffer mean (SD)
Day 1	25 wt% F-zirconia fillers	1.51 (0.09)	1.51 (0.07)	1.52 (0.20)
	50 wt% F-zirconia fillers	1.77 (0.03)	1.72 (0.06)	1.83 (0.21)
Day 3	25 wt% F-zirconia fillers	1.25 (0.01)	1.25 (0.03)	1.36 (0.13)
	50 wt% F-zirconia fillers	1.53 (0.02)	1.44 (0.04)	1.57 (0.28)
Day 7	25 wt% F-zirconia fillers	1.31 (0.10)	1.25 (0.01)	1.38 (0.12)
	50 wt% F-zirconia fillers	1.43 (0.03)	1.42 (0.02)	1.60 (0.20)
Day 14	25 wt% F-zirconia fillers	0.36 (0.00)	0.36 (0.01)	0.40 (0.05)
	50 wt% F-zirconia fillers	0.47 (0.01)	0.45 (0.02)	0.52 (0.72)
Day 28	25 wt% F-zirconia fillers	0.13 (0.01)	0.10 (0.00)*	0.12 (0.00)*#
	50 wt% F-zirconia fillers	0.15 (0.00)	0.12 (0.01)*	0.16 (0.02)*

Note: The superscript symbol * means statistical difference compared to that in distilled water, # means statistical difference compared to that in the neutral buffer (*p* < 0.05).

**TABLE 3:** Cumulative fluoride release (ppm) from the experimental composite resin with fluoride-doped nano-zirconia fillers in different buffers at each detection point during 28 days.

Time	Groups	Distilled water mean (SD)	Neutral buffer mean (SD)	Acidic buffer mean (SD)
Day 1	25 wt% F-zirconia fillers	1.51 (0.09)	1.51 (0.07)	1.52 (0.20)
	50 wt% F-zirconia fillers	1.77 (0.03)	1.72 (0.01)	1.83 (0.21)
Day 3	25 wt% F-zirconia fillers	4.01 (0.07)	4.00 (0.09)	4.23 (0.07)*
	50 wt% F-zirconia fillers	4.83 (0.06)	4.60 (0.08)	4.97 (0.49)
Day 7	25 wt% F-zirconia fillers	9.23 (0.34)	8.95 (0.15)	9.75 (0.53)*
	50 wt% F-zirconia fillers	10.55 (0.11)	10.28 (0.08)	11.36 (0.81)*
Day 14	25 wt% F-zirconia fillers	11.75 (0.34)	11.49 (0.18)	12.57 (0.87)
	50 wt% F-zirconia fillers	13.84 (0.15)	13.40 (0.15)	14.98 (1.27)*
Day 28	25 wt% F-zirconia fillers	13.60 (0.44)	12.94 (0.19)	14.18 (0.87)*
	50 wt% F-zirconia fillers	15.90 (0.16)	15.04 (0.05)	17.24 (1.48)*

Note: The superscript symbol * means statistical difference compared to that in distilled water (*p* < 0.05).

### 3.4 Antibacterial property

**FIGURE 4:**
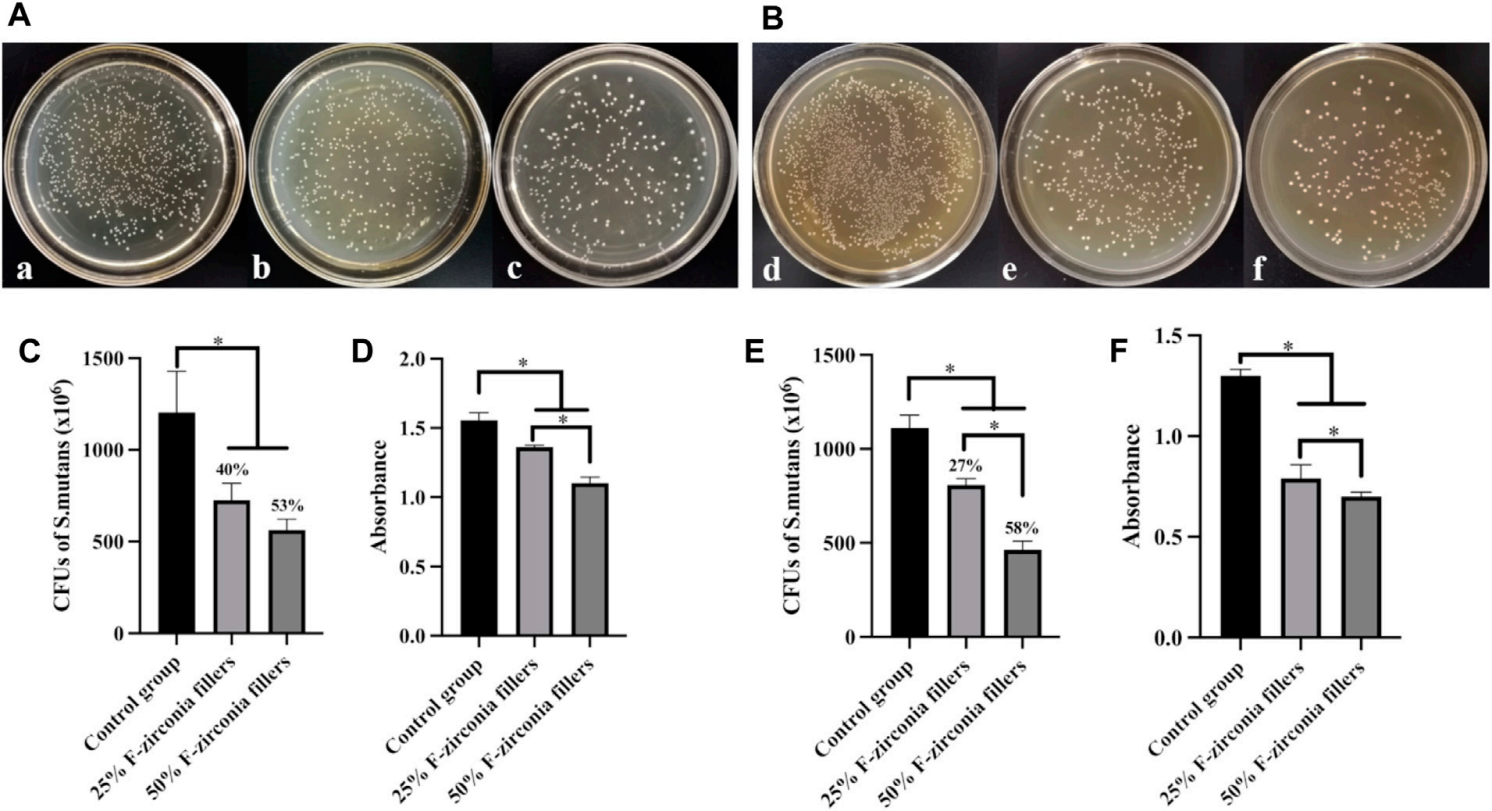
The result of antibacterial effect against S. mutans of the experimental composite resin with fluoride-doped nano-zirconia fillers [**(A,C,D)** Antibacterial effect against biofilms of S. mutans; **(B,E,F)** Antibacterial properties against planktonic bacteria; **(A,B)** Images of bacterial colonies; a,d, Control group; b,e, 25 wt% F-zirconia fillers; c,f, 50 wt% F-zirconia fillers; **(C,E)** Number of bacterial colonies (CFUs) and the antibacterial rates (AR); **(D,F)** The CCK-8 test; The symbol * means *p* < 0.05.).

Table 4 and Figure 4 showed the antibacterial effects of the experimental CRs against biofilms of S. mutans and the planktonic bacteria. In terms of the contact antibacterial effect against biofilms, the group of 50 wt% F-zirconia fillers showed better antibacterial activity with the antibacterial rate of 53%, followed by the group of 25 wt% F-zirconia fillers (40%), while the difference was not statistically significant (*p* ≥ 0.05). In terms of releasing antibacterial effect against the planktonic bacteria, the antibacterial rate improved from 27% to 58% with the increase of F-zirconia fillers, and the difference was statistically significant (*p* < 0.05).

**TABLE 4:** The antibacterial activities for the biofilm of S.mutans and planktonic bacteria evaluation.

Groups	Biofilm of S.mutans evaluation Log (CFU/mL)	Planktonic bacteria evaluation Log (CFU/mL)
Control group	9.08 ± 0.07	9.05 ± 0.02
25 wt% F-zirconia fillers	8.86 ± 0.05*	8.91 ± 0.01*
50 wt% F-zirconia fillers	8.75 ± 0.04*	8.66 ± 0.03*#

Note: The superscript symbol * means statistically different compared to the control group, # means statistically different compared to the 25 wt% F-zirconia fillers group (*p* < 0.05).

The metabolic activities of S. mutans after culturing with the experimental CRs in the CCK-8 test were also presented in Figures 4D, F. In general, with the amount of fluoride-doped nano-zirconia fillers increased, the absorbance of bacterial suspension was significantly lower than that of the control group (*p* < 0.05). Furthermore, the differences between two experimental groups observed in antibacterial effects against both biofilms and planktonic bacteria showed statistical significance (*p* < 0.05).

### 3.5 Aging test

#### 3.5.1 ΔE00 results

The CIELab values of the experimental CRs, which contained the L* (lightness), a* (red-to-green axis) and b* (yellow-to-blue axis) values, were summarized in Table 5. According to the result of one-way ANOVA and multiple comparisons, the control group showed significant differences in CIELab values as compared with another two groups both before and after the thermal aging procedure (*p* < 0.05). The difference of CIELab values between the experimental groups had no statistical significance (*p* ≥ 0.05). In addition, comparison of ΔE00 values during the thermal aging progress among three groups was also exhibited in Table 5. The control group had the highest ΔE00 value. ΔE00 values of the experimental groups were significantly lower than that of the control group (*p* < 0.05). And ΔE00 value of the group of 50 wt% F-zirconia fillers was lower than that of the group of 25 wt% F-zirconia fillers, but the difference had no statistical significance (*p* ≥ 0.05).

**TABLE 5:** ΔE00 during the thermal aging progress of the experimental composite resin with fluoride-doped nano-zirconia fillers.

Group	CIELab value (mean ± SD)			ΔE00 value (mean ± SD)
		T0	T1	
Control group	L*	38.28 ± 3.19	39.67 ± 3.00	1.69 ± 0.35
	a*	−1.04 ± 0.12	−1.24 ± 0.07	
	b*	7.03 ± 0.97	6.04 ± 0.64	
25 wt% F-zirconia fillers	L*	42.30 ± 2.03	43.20 ± 3.18	1.20 ± 0.17γ
	a*	1.52 ± 1.53	1.84 ± 1.98	
	b*	11.83 ± 6.08	12.38 ± 6.79	
50 wt% F-zirconia fillers	L*	45.28 ± 5.23	45.63 ± 5.19	0.78 ± 0.44γ
	a*	1.77 ± 1.18	1.74 ± 1.17	
	b*	11.91 ± 4.58	11.44 ± 4.50	

Note: The superscript symbol γ means statistical difference compared to the control group (*p* < 0.05).

#### 3.5.2 Microhardness

Microhardness values of the experimental CRs before and after the thermal aging process were presented in Table 6. The microhardness of the control group in T0 was 18.30 ± 1.25 HV0.1, while those of the groups of 25 wt% F-zirconia fillers and 50 wt% F-zirconia fillers were 48.50 ± 10.49 HV0.1 and 73.72 ± 5.64 HV0.1, respectively. The microhardness values of all three groups decreased after the thermal aging test (in T1). However, there were no statistically significant differences between the microhardness values of the same group before and after the thermal aging process (*p* ≥ 0.05). The statistical results revealed significant differences between the control and experimental groups in both T0 and T1 (*p* < 0.05). In addition, the variation of microhardness values (T0-T1) of each group was also analyzed here. And the difference of the variations among three groups was not statistically significant (*p* ≥ 0.05).

**TABLE 6:** Microhardness values of the experimental composite resin with fluoride-doped nano-zirconia fillers in different times during the thermal aging test.

Group	Microhardness value (mean ± SD, HV0.1)		
	T0	T1	T0-T1
Control group	18.30 ± 1.25	14.61 ± 0.97	3.69 ± 0.58
25 wt% F-zirconia fillers	48.50 ± 10.49*	44.38 ± 9.31*	4.36 ± 1.83
50 wt% F-zirconia fillers	73.72 ± 5.64*#	71.02 ± 3.56*#	2.69 ± 2.93

Note: The superscript symbol * means statistically different compared to the control group, # means statistically different compared to the 25 wt% F-zirconia fillers group (*p* < 0.05).

### 3.6 Cytotoxicity

**FIGURE 5:**
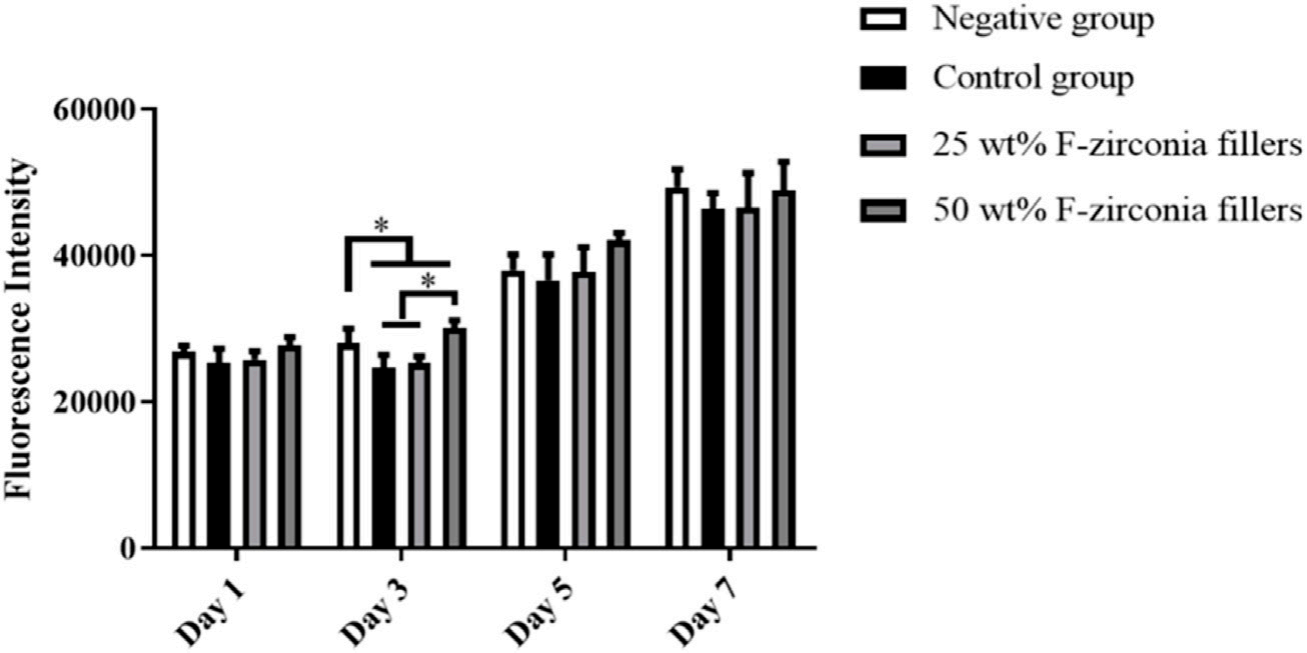
Fluorescence intensities of HDPs at 1, 3, 5 and 7 days in response to eluates of the experimental composite resin with fluoride-doped nano-zirconia fillers in the alamar blue assay (The symbol * means *p* < 0.05).

The alamar blue assay was applied in this study to verify the cytotoxicity of the experimental CRs to human dental pulp cells (HDPs). Fluorescence intensities produced by healthy HDPs at 1, 3, 5 and 7 days in response to eluates of the control group or experimental groups were presented in Figure 5. Statistical results with one-way ANOVA analysis revealed that the viabilities of HDPs exposed to eluates of each group were not significantly different from that of the negative group (*p* ≥ 0.05). But fluorescence intensities of cells exposed to elutes at day 3 were slightly lower than that of the negative group (*p* < 0.05).

## 4 Discussion

Dental composite resins (CRs) are the most widely used direct restorative materials in clinic because of their high mechanical strength and aesthetic superiority. However, as the traditional CRs has little resistance to bacterial infection, which was considered as the major cause of dental caries, secondary caries might occur adjacent to the CRs restoration margins and shorten their lifespans ultimately. Thus, the development of antibacterial CRs is one of the most important investigations regarding novel dental materials.

The antibacterial action of fluoride agents against cariogenic bacteria has been widely appreciated by previous studies. Fluoride ions in saliva can diffuse into bacterial cells in the form of HF, where they decompose into both hydrogen ions and fluoride ions. This process can not only inhibit the action of enzymes directly but also stimulate more HF to diffuse into the cells (Hamilton, 1977). In addition, the increase of hydrogen ions within the cells can reduce bacterial acid production (Hamilton and Ellwood, 1978). Given the definite antibacterial effect of fluoride agents, various fluoride-releasing restorative materials have emerged and were considered as fluoride reservoirs, which might increase the fluoride level locally (Wiegand et al., 2007; Wei Su et al., 2019). To date, addition of fluoride-containing inorganic fillers was considered as the major method to develop novel fluoride-releasing CRs. Both the soluble salts such as calcium fluoride (CaF2) and the slightly soluble salts like ytterbium fluoride (YbF2) and fluoro-alimino-silicate glasses (FAG) had been applied in previous studies. However, it was reported that most of them showed a “burst release” of fluoride ions and the dissolution of fluoride agents had an adverse effect on the mechanical properties (Van der Laan et al., 2019). It is not hard to spot that those inorganic fluoride agents were added by mixing with other fillers and there were only physical compatibilities among all particles, without chemical combination. Thus, the dissolution of fluoride agents resulted in porous structure of the CRs, which might damage the dense structure of the materials and furtherly declined the strength. It is necessary to improve the chemical structure of inorganic fillers to replace the way of adding fluoride agents directly. Cheng et al. produced a kind of core-shell nanofibers containing sodium fluoride (NaF) and used as fillers of CRs (Cheng et al., 2014). Results showed that fluoride releasing with minor burst release could be achieved, which was quite superior to the case of adding NaF nanocrystals directly. Similarly, a novel LiAl-F layered double hydroxide (LDH) was also developed by Su et al. and was supposed to be a fluoride reservoir filler for CRs (Wei Su et al., 2019). However, this kind of researches were still in the early stage of exploration and were expected to be carried on furtherly.

Although the best way of developing fluoride-releasing CRs has not yet been concluded, the previous studies suggested that it was of necessity to develop a novel CRs with efficient fluoride-releasing effect and proper mechanical properties. Recently, nano-zirconia particles have been used as the reinforcing fillers in dental CRs (Hong et al., 2020). Zirconium salts, which were a kind of raw compositions for the synthesis of zirconia particles, have been proven to exhibit strong chelate formation characteristics. It could form coordination bonds with multiple fluoride ions, forming a highly efficient “fluoride ions receptors” (David et al., 2002). For this reason, zirconium salts were frequently used for fluoride removal in drinking water (Velazquez-Jimenez et al., 2014). Burgess et al. introduced a novel monomer with zirconium fluoride chelate and confirmed it could also be applied to dental resin-based materials (Xu et al., 2006). In our preliminary study, a kind of novel CR loaded with fluoride-doped nano-zirconia (F-zirconia) particles was prepared. The result showed that the major X-ray diffraction (XRD) pattern of fluoride-doped nano-zirconia particles changed from the tetragonal phase to the monoclinic phase with the increase of fluorine content (Zheng et al., 2021). Similarly, in the process of plasma fluorination of yttrium stabilized zirconia, Wolter et al. (2011) observed that the content of oxygen atoms gradually decreased with the increase of fluorine atoms in X-ray photoelectron spectroscopy, which indicated that fluorine atoms might replace oxygen atoms to occupy the spatial position in zirconium dioxide crystal cells. Hence, based on the uniform distribution of fluorine in the EDS elemental maps, we inferred that the change of XRD pattern was also caused by the substitution of oxygen atoms by fluorine atoms. Fluoride ions could release from the nanoparticles in ddH2O and showed proper antibacterial effect.

Furtherly, the effect of pH values on fluoride release and the action mode of antibacterial activities were explored in the study. In addition, the mechanical performance and aging properties, as well as the cytotoxicity of the experimental CR were also evaluated here.

Previous studies revealed that addition of nano-zirconia fillers could improve the mechanical strength of CRs, but might also reduce the transparency. When the content of nano-zirconia fillers reached 55 wt%, the CRs could not be cured completely through light-curing (Hong et al., 2020). Thus, we prepared two groups of experimental CRs loaded with 25 wt% or 50 wt% F-zirconia fillers, respectively. Figure 1 showed the SEM micrographs of the experimental CRs, and it could be seen that nano-fillers distributed well over the whole surface of resin matrix. Good dispersibility of fillers was attributed to the mechanical strength of CRs (Hata et al., 2022). As presented in Table 1, the three-point bending test showed that the addition of F-zirconia fillers could maintain the flexure strength of experimental CRs. The flexure strengths of all three groups of CRs were >80 MPa, which could meet to the ISO 4049 standard (Goracci et al., 2014). Furtherly, compared to the control group, addition of F-zirconia fillers could significantly improve the wear resistance of experimental CRs. With the increase of F-zirconia fillers, height loss in the surface wear zones decreased significantly and the wear zones were relatively flat (Table 1; Figure 2).

The daily and cumulative amounts of fluoride release from the experimental CRs were presented in Table 2 and Table 3. In general, the two groups of experimental CRs showed continuous fluoride release within 28 days, and the fluoride release was positively correlated with the content of F-zirconia fillers. The significantly higher amounts of fluoride release were observed during the first 7 days. And the daily amounts of fluoride release decreased in day 14 and 28. It was considered that the fluoride ions were dissolved from the surface of specimens in the early stage. Subsequently, a longer time was required for the fluoride ions to diffuse from the inner part of specimens. The trend of fluoride release here was consistent with that in other similar studies (Naoum et al., 2013; Paul et al., 2020). Compared with the traditional GICs, which contained a large amount of fluoro-alimino-silicate glasses fillers, the experimental CRs showed lower fluoride release, relatively. As we all know that GICs had been widely used as dental restorative material in the past because of its excellent fluoride-releasing and anti-cariogenic properties. But studies have reported that GICs exhibited poor mechanical strength and undesirable wear resistance, which was closely related to the dissolution of soluble fillers (Moshaverinia et al., 2024). In fact, the principle of fluoride release from GICs was acid-base reaction in saliva. The reaction was rapid and uncontrolled, which often showed a “burst release” effect and furtherly led to obvious cracks of materials (Thongsri et al., 2024). In contract, the release of fluoride ions in cured CRs was achieved through diffusing outward when water penetration into the gap between the resin matrix and inorganic fillers. We compared the SEM images of the experimental CRs before and after fluoride release examination in the preliminary study (Zheng et al., 2021). And there were no holes or cracks appeared on the surface of the CRs after fluoride release examination. Thus, the release process of fluoride ions did not affect the structure of nano-zirconia particles, so that it did not damage the mechanical properties of the experimental CRs. Moreover, it was reported that only 0.03 ppm–0.07 ppm fluoride ions could be contributed to transform teeth demineralization to the remineralization phase (Ahmed et al., 2015). Moreover, Marquis R.E. suggested that fluoride ions could inhibit oral bacteria when the concentration reached 0.1 mM (Marquis, 1995). Thus, all observations during 28 days were within the expected range.

Meanwhile, the influence of various pH values on the release of fluoride ions from the experimental CRs was also investigated here. It was discussed that fluoride might interfere with the dynamics of dental caries process *in vivo*, so *in vitro* models that could simulated the caries process were recommended to test the effects of fluoride-releasing materials (Serra and Cury, 1992; Cury et al., 2016). One of the most important causes of dental caries is the dissolution of acids produced by cariogenic bacteria (Zhou et al., 2012). And it was reported that the incidence of caries increased significantly when pH value dropped to 4.0–5.5 (Di Lauro et al., 2023). Thus, it is of necessity to verify the fluoride release property of the experimental CRs in acidic media. As shown in Table 3 and Figure 3, it was interesting to find that the amount of fluoride release in the deionized water (ddH_2_O) was higher than that in the neutral buffer (pH 7.0). Like in previous studies, ddH_2_O was used as an accurate model of fluoride release from dental materials, and a reduced amount of fluoride release could be found when using neutral buffer or artificial saliva (Behrend and Geurtsen, 2001; Harhash et al., 2017). The reason for this was likely to be the multi-ionic environment accelerated the equilibration of fluoride ions. It could also be found that the amount of fluoride release increased in the acidic buffer (pH 4.2) in this study, but the change was not significant. Therefore, the result revealed that intraoral fluoride release from the experimental CRs might be enhanced on restorations or caries surfaces that were eroded by plaque-associated acids.

Among various of bacterial species in the oral environment, S. mutans was proven to play a major role in the formation of plaque biofilms and the development of caries (Sungurtekin-Ekci et al., 2015). Therefore, antibacterial effects of the experimental CRs were investigated against S. mutans biofilms on the surfaces, as well as the planktonic bacteria. The results of antibacterial effect were shown in Table 4 and Figure 4. Compared with the control group, two experimental groups showed obvious decrease of S. mutans colonies in the CFUs counting. Furtherly, the result of CCK-8 assay confirmed that the experimental CRs effectively inhibited the metabolic activities of S. mutans, and the antibacterial effect was enhanced with the increase of fluoride-doped nano-zirconia fillers content. In fact, a previous research suggested that the antibacterial effect of fluoride agents occurred by the diffusion of fluoride ions (Whitford et al., 1977). Nonetheless, the result here indicated that the experimental CRs could inhibit the growth of S. mutans not only by releasing of the fluoride ions in the media but also through direct contact with the biofilms on the surfaces. The large surface-to-volume ratio of nanoparticles on the surfaces of specimens could help to inhibit the bacterial adhesion and biofilm formation (Chladek et al., 2023). Hence, the antibacterial effect of the experimental CRs might be result from the synergistic effect of fluoride ions and the influence of nanoparticles. Moreover, as the CRs wearing out slowly during the chewing movement, more fluoride-doped nano-zirconia fillers shall be exposed to the oral environment and show antibacterial effect continuously.

To date, although CRs exhibit excellent aesthetic effects compared to other restorative materials, the aging of CRs is still inevitable. The aging process can not only lead to discoloration and poor esthetic result, but also affects clinical longevity and is an important cause of restoration failure with CRs (Chen et al., 2024). Thermal changes were thought to exacerbate the aging process, as the intraoral temperature changes constantly in line with breathing and eating. Thermal cycling was the most common approach that applied to simulate the aging of dental materials during temperature fluctuations that occur in the mouth in previous studies (Gale and Darvell, 1999; Tavas et al., 2023). It was carried out at temperatures equivalent to the intraoral temperature, ranging from 5 °C to 55 °C, and 10,000 cycles might be equivalent to 1 year (Gonder and Fidan, 2022). Color change (ΔE00 value) and microhardness of the experimental CRs affected by aging were presented in Table 5 and Table 6. The color stability of restoration materials was important to meet the esthetic demands in dentistry. It was reported that ΔE00 values between 0.8 and 1.8 were considered acceptable for color changes in clinic that could be detected by the human eyes (Paravina et al., 2015). Based on the results in this study, the control group and the experimental groups had the average ΔE00 values of 1.69, 1.20 and 0.78, respectively, which were all within the acceptable range. The control group showed the highest ΔE00 value. The resin matrix, especially the TEGDMA monomer, might be the major reason for color change of the experimental CRs. It was indicated that TEGDMA exhibited high hydrophilicity and interfere with the color stability by allowing more water diffusion (Fidan and Yağci, 2023). Previous research found that the amount and size of inorganic fillers affected the color stability of CRs obviously. On the one hand, CRs loaded with high amounts of fillers showed higher color stability than that loaded with less fillers. On the other hand, addition of nanoparticles could be advantageous to reduce the polymerization shrinkage of CRs by lowering the resin-to-filler ratio significantly (Hamdy, 2021). As a result, addition of the fluoride-doped nano-zirconia fillers with small sizes could reduce the color change of experimental CRs. However, given that the surface roughness is also vital for the esthetic effect of CRs, additional trials are necessary to observe and analysis the changes of microstructure with aging furtherly.

Moreover, it was observed that the microhardness values of all specimens decreased slightly after the thermal aging progress. This might be the result of cracks in the cross-linking of resin structure and the weak bonding between the matrix and fillers. But the variation of microhardness values among three groups was not statistically different.

As we all know, the restorative procedure was usually associated with loss of significant amounts of hard tissues due to caries attack. Application of composite resin following caries removal would contribute to potential adverse effects on the pulp tissue. Jiang et al. (2017) studied the impact of various dentin thicknesses on the cytotoxicity of three commercial restorative materials *in vitro* and showed that one of the materials reached cytotoxic levels when the thickness was at 1 mm. It is of significance to evaluate the cytotoxicity with cells in pulp tissue. As such we chose human dental pulp cells (HDPs) in cell culture here like previous studies (Schneider et al., 2019; Hadjichristou et al., 2020). Statistical results as showed in Figure 5 revealed that the viabilities of HDPs exposed to eluates of the experimental CRs were not significantly different from that of the negative group. Therefore, the experimental CRs developed here showed no significant cytotoxicity and was qualified for application in clinic.

There are still some limitations in this study. Given the anti-caries effects of fluoride ions include both inhibition of the cariogenic bacteria and remineralization of dentin or enamel, additional investigations are indeed required to clarify the remineralization of the novel CRs. In addition, the experimental conditions can not fully simulate the oral environments, which are also affected by the washing process of saliva and the effect of enzymes. Thus, further *in vivo* tests are needed before clinic application.

## 5 Conclusion

In summary, a novel composite resin loaded with fluoride-doped nano-zirconia fillers was developed successfully. The fluoride ions released continuously from the experimental CRs resulted in effective contact and releasing antibacterial properties. Addition of fluoride-doped nano-zirconia fillers could improve the color stability, wear resistance and microhardness of the experimental CRs, without reducing the flexure strength. Moreover, the novel CRs showed no cytotoxicity to HDPs. Thus, considerations can be made to use this kind of fluoride-releasing composite resin loaded with fluoride-doped nano-zirconia fillers to improve clinical treatment when the antimicrobial benefits are desired.

## Data Availability

The original contributions presented in the study are included in the article/supplementary material, further inquiries can be directed to the corresponding authors.
